# Utilizing maternal autoantibody patterns to predict risk of autism and intellectual disability in offspring

**DOI:** 10.21203/rs.3.rs-8929366/v1

**Published:** 2026-05-07

**Authors:** Judy Van de Water, Katelien Blumenthal, Stacey Alexeeff, Lauren Weiss, Robert Yolken, Paul Ashwood, Lisa Croen, Joseph Schauer

**Affiliations:** University of California, Davis; University of California, Davis; Kaiser Permanente Division of Research; Department of Psychiatry and Institute for Human Genetics, University of California, San Francisco; Johns Hopkins University School of Medicine; Kaiser Permanente Division of Research; University of California, Davis

## Abstract

Maternal autoantibody-related autism (MARA) is a subset of autism in which specific patterns of maternal autoantibodies (aABs) in the circulation of pregnant women have been associated with increased autism risk in offspring. While initial MARA studies identified patterns consisting of two maternal aABs that predicted increased risk of autism, other multi-aAB patterns predictive of autism and other neurodevelopmental disorders have not yet been fully assessed. In this study, we aimed to determine if additional patterns of MARA aABs can be used to predict the risk of autism and intellectual disability (ID). We tested maternal plasma samples from the Early Markers for Autism (EMA) study for reactivity to eight proteins with clinical relevance in our initial MARA studies. Least Absolute Shrinkage and Selection Operator (LASSO) statistical modeling was used to identify patterns of three maternal aABs that were predictive of offspring autism and ID risk. We identified novel patterns consisting of three aABs associated with increased risk of autism or ID compared to general population controls (GP). Additionally, we found that specific patterns of three maternal aABs differentially predicted risk of autism with intellectual disability (AU ID) and autism without intellectual disability (AU noID), compared to GP. Overall, novel patterns consisting of three maternal aABs have been identified and can be used to predict child clinical risk of autism, ID, and autism subgroups, AU ID and AU noID.

## Introduction

Neurodevelopmental disorders (NDD) are a class of disorders characterized by deficits in learning, language, behavior, or physical function (1). Fetal and early life genetic and environmental factors converging on immune responses can impact offspring development. Several studies have shown that dysregulation of the maternal immune system contributes to NDD, including autism (AU), attention deficit hyperactivity disorder (ADHD), and developmental delay (DD) (2, 3). Throughout pregnancy, maternal immunoglobulin G (IgG) is transported across the placenta from maternal to fetal circulation to provide immune protection to the fetus (4). While most transported antibodies provide protective immunity, pathogenic autoantibodies (aABs) can also be passed across the placenta during pregnancy (4, 5). Maternal aAB transport to fetal circulation has been associated with offspring neonatal autoimmune diseases as well as NDDs, including AU, ADHD, ID, and learning disorders (4–10). Previously, we demonstrated that specific aABs found in maternal circulation were associated with an AU diagnosis in offspring (11–13). We identified the aAB targets as eight different proteins, including lactate dehydrogenase A + B (LDHA + B), stress induced phosphoprotein-1 (STIP1), collapsin response mediator protein 1 + 2 (CRMP1 + 2), guanine deaminase (GDA), neuron specific enolase (NSE), and Y-box binding protein 1 (YBOX) (12, 13). These proteins can all be found in the developing brain and, therefore, may be targets for altered neurodevelopment.

In a more recent study, we identified specific patterns of maternal aABs that were associated with an increased risk for an AU diagnosis in offspring (6). This subset of AU is termed maternal autoantibody-related autism (MARA). Using postnatal maternal plasma samples from the Childhood Autism Risks from Genetics and the Environment (CHARGE) study (14), we identified nine MARA aAB patterns, including CRMP1 + CRMP2, STIP1 + NSE, CRMP2 + STIP1, YBOX+LDHB, YBOX + GDA, CRMP1 + STIP1, YBOX+LDHA, CRMP1 + GDA, and YBOX+STIP1 that were predictive of MARA (6). In a subsequent study, prenatal maternal plasma samples from the Early Markers for Autism (EMA) study were used to examine the presence of these aAB patterns during gestation, and to determine which patterns could predict risk of AU, intellectual disability (ID), autism with ID (AU ID), and autism without ID (AU noID) (7). Further, to understand the pathological impact of MARA-aABs on offspring neurodevelopment, we developed preclinical rodent models that replicated the gestational exposure to specific patterns of the MARA-aABs (15–17). These studies demonstrated that the MARA-specific aAB patterns impacted offspring behavior and brain development. We aim to expand upon the prior studies to extend the repertoire of known maternal aAB patterns by determining if there are distinct combinations of three aABs associated with an increased risk of AU, ID, AU ID, and AU noID.

## Methods

### Study subjects

The Early Markers for Autism (EMA) study is a population-based case-control study designed to investigate environmental, genetic, and immune factors that influence offspring risk for autism and other developmental disorders (18). Archived maternal blood samples collected between March 2000 and July 2003 from pregnant individuals who participated in the prenatal extended alpha-fetoprotein screening program (XAFP) were retrieved. Participants were from urban, suburban, and rural areas with multicultural backgrounds in Southern California. Children born to these individuals who were receiving services from a California Department of Developmental Services Regional Center for autism (AU, n = 540) or intellectual disability (ID, n = 184) were identified. General population controls (GP, n = 420) age- and sex-matched to AU cases were randomly selected from birth certificate files. GP controls had no record of Regional Center service receipt.

### Diagnostic verification

Diagnostic criteria and verification of AU and ID diagnoses was described in a previous publication (18).Briefly, Regional Center records were obtained and reviewed using a standard form by an expert clinician. AU diagnoses were confirmed in accordance with the DSM-IV-TR criteria. ID was defined as cognitive scores < 70 on all standardized cognitive and functional tests. Children with AU were further classified based on the presence of an intellectual disability diagnosis: autism with intellectual disability (AU ID, n = 285) or autism without intellectual disability (AU noID, n = 219).

### Specimen collection and preparation

Maternal blood was collected at 15–20 weeks of gestation in citrate dextrose (BD Diagnostic, Franklin Lakes, NJ). Plasma was separated, labeled, and stored at −80°C. For use in experiments, plasma was thawed at room temperature, vortexed, and centrifuged at 13 000 RPM for 10 minutes.

### Enzyme Linked Immunosorbent Assay (ELISA)

ELISA was performed to test the reactivity of maternal plasma aABs to eight MARA protein antigens as previously described (6). The protein concentration and plasma sample dilutions were optimized. Briefly, 96-well plates were coated with 100 μL of protein (1.5–3 μg/μL) in carbonate coating buffer (pH 9.6) overnight at 4°C. Plates were washed four times with 0.05% Phosphate-Buffered Saline Tween-20 (PBST) and blocked with 2% Super Block (Thermo Scientific, Rockford, IL, USA) for 30 minutes at room temperature. Plasma samples were diluted 1:250–1:1000 in sample diluent (0.05% PBST + 0.2% Super Block). After blocking, 100 μL of the diluted plasma samples were added to their respective wells and incubated for 1.5 hours. Samples were washed four times with 0.05% PBST and incubated with goat anti-human IgG-horseradish peroxidase (HRP) (Kirkegaard & Perry Laboratories, Inc., Gaithersburg, MA, USA) diluted 1:10 000 in sample diluent for 1 hour at room temperature. Plates were washed four times with 0.05% PBST. For colorimetric detection, 100 μL of BD optEIA liquid substrate for ELISA consisting of hydrogen peroxide and 3,3’, 5,5’ tetramethylbenzidine (TMB) (BD Biosciences, San Jose, CA, USA) was added to the plates for 4 minutes at room temperature. The reaction was stopped with 50 μL of 2 N HCl. Sample absorbance was measured at 450 nm on an iMark Microplate Absorbance Reader (Biorad, Hercules, CA, USA).

### Statistical analysis

Least Absolute Shrinkage and Selection Operator (LASSO) modeling was used to identify maternal aAB patterns that were predictive of offspring AU or ID risk. LASSO is a machine learning model that selects the most relevant set of predictors in a high-dimensional dataset by using a penalty term to shrink less relevant coefficients exactly to zero (19). We fit two multinomial LASSO models with three outcome groups: the first model included outcomes AU, ID, and GP, and the second model included outcomes AU ID, AU noID, and GP. Because LASSO uses penalization to shrink many coefficients exactly to zero and does not conduct inference on associations or rely on conventional maximum-likelihood estimation, standard errors and confidence intervals are not directly available from the model and are therefore not reported. LASSO models included all patterns of three maternal aABs for potential selection by the model. LASSO models were fit using package glmnet in R version 4.5.1 (20).

## Results

### Patterns consisting of three maternal aABs associated with increased AU risk compared to GP

The LASSO model comparing AU, ID, and GP identified seventeen patterns of three aABs which were associated with increased risk of AU compared to GP (Table 1). The strongest AU-specific aAB patterns were CRMP1 + CRMP2+LDHA, CRMP1 + STIP1+LDHA, CRMP1 + GDA+LDHA, YBOX + GDA+LDHA, STIP1 + NSE + GDA, YBOX+LDHA+LDHB and CRMP2 + STIP1+LDHB. Each of those patterns was present in 2 to 8 AU cases and not present in any ID case or GP control. LASSO ORs for those patterns ranged from 3.07 to 5.30 (Table 1). Additional patterns include NSE+LDHA+LDHB, NSE+LDHA + GDA, NSE+LDHB + GDA, CRMP2 + NSE+LDHA, CRMP2 + GDA+LDHB, CRMP1 + CRMP2+YBOX, CRMP1 + CRMP2+LDHB, CRMP2 + STIP1+LDHA, YBOX + GDA+NSE, and YBOX + GDA+LDHB. LASSO ORs for these patterns ranged from 1.09 to 1.95 (Table 1).

### Patterns consisting of three maternal aABs associated with increased ID risk compared to GP

Using LASSO modeling with AU, ID, and GP as outcome groups, we identified three patterns consisting of three maternal aABs that were associated with an increased risk of ID compared to GP (Table 1). The first pattern associated with an ID-specific risk is YBOX+LDHA + NSE, occurring in the ID group, but not AU or GP groups (ID vs. GP: OR 8.80) (Table 1). An additional two patterns, CRMP1 + NSE+LDHB (ID vs. GP: OR 9.52) and YBOX+LDHA+STIP1 (ID vs. GP: OR 2.47), confer specificity for ID diagnosis, occurring primarily in the ID group but a low frequency of the AU group and none of the GP group (Table 1).

#### Patterns consisting of three maternal aABs similarly associated with AU and ID risk compared to GP

LASSO modeling with three outcome groups (AU, ID, GP) identified patterns that were predictive of risk of both AU and ID when compared to GP. CRMP2 + STIP1+GDA was predictive of an increased risk of AU status or ID status compared to GP (AU vs. GP: OR 1.26; ID vs. GP: OR 1.29) (Table 1). LDHA+LDHB + GDA was predictive of a decreased risk of AU and ID compared to GP (AU vs. GP: OR 0.64; ID vs. GP: OR 0.64) (Table 1).

#### Patterns consisting of three maternal aABs associated with increased risk of AU ID and AU noID compared to GP

AU and ID are distinct NDD, yet the two diagnoses can often co-occur (21). To examine the differential effects of maternal aAB patterns on AU ID and AU noID, three outcome groups were defined for LASSO modeling (AU ID, AU noID, GP). Thirteen AU-specific patterns consisting of three maternal aABs were identified; nine of these patterns were AU subtype specific. Five out of nine of these patterns occurred primarily in the AU ID subgroup: CRMP1 + GDA+LDHA (AU ID vs. GP: OR 2.94), CRMP2 + GDA+LDHB (AU ID vs. GP: OR 2.86), CRMP1 + STP1+NSE (AU ID vs. GP: OR 1.15), CRMP1 + NSE+LDHB (AU ID vs. GP: OR 1.69), and CRMP1 + CRMP2+YBOX (AU ID vs. GP: OR 1. 69) (Table 2). Four out of nine of these AU-specific patterns occurred predominantly in the AU noID subgroup: STIP1 + NSE+LDHB (AU noID vs. GP: OR 4.75), YBOX + GDA+NSE (AU noID vs. GP: OR 2.40), YBOX+LDHA + GDA (AU noID vs. GP: OR 2.40), and YBOX+LDHB + GDA (AU noID vs. GP: OR 2.40) (Table 2). The patterns CRMP1 + CRMP2+LDHA, CRMP1 + STIP1+LDHA, CRMP1 + STIP1+LDHB, and YBOX+LDHA+LDHB were AU-specific patterns that were not skewed towards an AU subgroup (Table 2).

## Discussion

This study is the first to identify patterns of three maternal aABs during pregnancy that predict risk of AU, ID, and AU subgroups in a prenatal sample. We found patterns consisting of three maternal aABs in the blood of pregnant females that are AU- and ID-specific. These patterns are unique from the two aAB patterns previously associated with MARA, and can be utilized in clinical tests to increase risk prediction specificity for AU, ID, and AU subgroups.

The first set of AU-specific patterns NSE+LDHA+LDHA, NSE+LDHA + GDA, NSE+LDHB + GDA, CRMP2 + NSE+LDHA, and CRMP2 + GDA+LDHB are novel–they do not include any previously identified two aAB MARA patterns–and expand the repertoire of patterns that can be used to predict risk of AU diagnosis. NSE is an enzyme that regulates neuronal survival and can regulate neurite outgrowth (22). Neuroinflammation and neurodegeneration are associated with altered NSE levels and function (22), indicating a potential for aAB-related pathogenesis during neurodevelopment with respect to NSE targeting. In addition, GDA, or “cypin” in the brain, is a neuroprotective enzyme that regulates neuronal plasticity via dendritic spines (23). Alteration to the neuroplastic and neuroprotective functions of GDA can be associated with aberrant neurodevelopment. LDHA is a metabolic enzyme expressed by cells throughout the brain, supporting proper neuronal and glial cell functioning (24). Disruption to LDHA could therefore contribute to neuronal dysregulation, exacerbating skewed neurodevelopmental functioning. In addition, LDHB maintains similar functions in neurodevelopment to that of LDHA. Dysregulation of this enzyme could similarly lead to skewed neuronal functioning and has been associated with impaired memory (24, 25). Finally, CRMP1 is an intracellular protein expressed by neurons in the embryonic and postnatal brain throughout development (26). More specifically, CRMP1 is important for neurite outgrowth and growth cone collapse, processes critical for proper axon guidance and dendrite formation (26, 27). Dysregulation of CRMP1 has been shown to lead to impaired axon outgrowth and dendritic spine and synapse formation, altering the morphology and function of neurons in the developing brain (28). Altogether, a combination of aABs targeting enzymes and proteins important for proper neuronal functioning may be driving the altered neurodevelopmental phenotypes observed in autistic offspring. Twelve out of seventeen of the AU-specific maternal aAB patterns are further refinements of the initial two aAB MARA patterns identified (6, 7). CRMP1 + GDA+LDHA and CRMP1 + STIP1+LDHA contain previously identified patterns, CRMP1 + GDA and CRMP1 + STIP1, respectively (6, 7). For both patterns, the addition of LDHA increases the specificity of these patterns for predicting AU-specific risk. Similarly, YBOX + GDA+NSE contains the pattern YBOX + GDA (7). The addition of NSE to this pattern increases pattern specificity for AU, as it only occurred in the AU group. YBOX is an intracellular protein that regulates transcription and translation. In the brain, YBOX is found primarily in the embryonic and early postnatal brain within the cytoplasm and dendrites of neuronal cells (29). Therefore, the combination of aABs targeting YBOX, GDA, and NSE may be targeting early neuronal pathways critical for optimal cognitive and behavioral functioning. Additionally, CRMP2 + STIP1+LDHA and CRMP2 + STIP1+LDHB both contain the previously identified pattern CRMP2 + STIP1 (6, 7). While this two-analyte aAB pattern was found in a small percentage of the ID group (7), the addition of aABs against LDH proteins increases the pattern specificity for predicting AU diagnosis. YBOX+LDHA+LDHB, YBOX + GDA+LDHA, and YBOX + GDA+LDHB all contain a combination of the patterns YBOX + GDA, YBOX+LDHA, and YBOX+LDHB. While alone these two aAB patterns are sufficient to predict risk of AU, the three-way aAB combinations have increased specificity for predicting risk of AU over the two-way patterns (7). STIP1 + NSE + GDA contains the two-way pattern STIP1 + NSE, and CRMP1 + CRMP2+YBOX, CRMP1 + CRMP2+LDHA, and CRMP1 + CRMP2+LDHB patterns all contain the two-way pattern CRMP1 + CRMP2 that predict an increased AU risk. While STIP1 + NSE and CRMP1 + CRMP2 alone strongly predict risk of AU, a third aAB may contribute to the subgroup specification of AU diagnosis.

We found that three different patterns consisting of three maternal aABs were associated with an ID-specific risk, including CRMP1 + NSE+LDHB, YBOX+LDHA + NSE, and YBOX+LDHA+STIP1. Interestingly, the CRMP1 + NSE+LDHB pattern is completely novel–there are no two-analyte aAB combinations targeting these proteins that predict risk of ID or AU. The presence of maternal aABs targeting a combination of CRMP1, LDHB, and NSE proteins may promote early dysregulation pathways critical for proper neuronal development and overall learning and memory. The pattern YBOX+LDHA + NSE predicted risk of an ID diagnosis. While previous studies identified that the pattern YBOX+LDHA primarily predicts risk of AU diagnosis (7), with the addition of NSE this pattern becomes specific for predicting risk of an ID diagnosis. The third pattern that predicts risk of ID is YBOX+LDHA+STIP1. YBOX+LDHA and STIP1 + YBOX were previously identified as two aAB patterns that primarily predict risk of AU diagnosis (6, 7), while the combination STIP1 + LDHA does not predict the risk of AU or ID. However, when a pattern of three aABs are combined, the YBOX+LDHA+STIP1 pattern becomes specific for ID. We hypothesize that when all three proteins are targeted, they lead to pathways skewing cognitive development and intellectual functioning.

Children with co-occurring AU and ID present both autistic behaviors as well as lowered cognition scores (21, 30). Studies have investigated structural differences in the brains of children with AU ID and have found regional differences in the white and grey matter properties in these children compared to children with ID only (31). Therefore, it is important to understand how specific patterns of maternal aABs can influence the risk of AU ID and AU noID. We found nine patterns consisting of three maternal aABs associated with increased autism risk that differed based on the presence or absence of an additional ID diagnosis. Patterns consisting of CRMP1 + GDA+LDHA, CRMP2 + GDA+LDHB, CRMP1 + STIP1+NSE, CRMP1 + CRMP2+YBOX, and CRMP1 + NSE+LDHB occurred primarily in the AU ID group. CRMP2 + GDA+LDHB and CRMP1 + NSE+LDHB are completely novel patterns that predict risk of AU ID. Additionally, while CRMP1 + NSE+LDHB is a pattern that predicts risk of ID alone, it can also be used to predict risk of the AU ID subgroup with an AU diagnosis. On the other hand, CRMP1 + GDA+LDHA contains the two aAB pattern CRMP1 + GDA, a preexisting two aAB pattern that predicted risk of AU ID (7). The addition of LDHA also skews the pattern to be primarily associated with AU ID diagnosis. Similarly, the pattern CRMP1 + STIP1+NSE contains two aAB patterns CRMP1 + STIP1 and STIP1 + NSE which are preexisting patterns that predict risk of AU (6). While STIP1 + NSE does not indicate an additional ID diagnosis, CRMP1 + STIP1 results primarily in AU noID outcome (7). However, a combination of all three aABs enhances this specificity for predicting risk of AU ID diagnosis. These combinations may promote the phenotype of these comorbidities through cognition and learning related pathways. Finally, CRMP1 + CRMP2+YBOX consists of the two-analyte aAB pattern CRMP1 + CRMP2 which accurately predicts AU with no skewedness towards an ID diagnosis (6, 7). The addition of YBOX to this pattern shifts the diagnosis to AU ID, indicating that targeting YBOX may have additional impacts on intellectual functioning and development.

While some patterns confer susceptibility for comorbid AU and ID, STIP1 + NSE+LDHB, NSE+YBOX + GDA, YBOX+LDHA + GDA, and YBOX+LDHB + GDA occurred primarily in the AU noID group. In the pattern STIP1 + NSE+LDHB, addition of LDHB to the previously identified STIP1 + NSE pattern confers specificity for AU noID diagnosis, indicating the presence of differential pathways targeted by multi-aAB combinations, leading to phenotypic differences under the autism umbrella. Similarly, AU noID patterns YBOX + GDA+NSE, YBOX + GDA+LDHA, and YBOX + GDA+LDHB all contain the YBOX + GDA pattern, which is specific for AU noID (7). In addition, YBOX+LDHA and YBOX+LDHB are both patterns that predict risk for AU; however, neither predicts risk of an AU subgroup. Therefore, it is likely that the aAB combination YBOX + GDA is sufficient to skew towards an AU noID subgroup.

Overall, these findings indicate that patterns of three maternal aABs can be used to predict AU and ID risk with greater specificity than some previous patterns consisting of two aABs. By identifying these patterns, we can assess how in utero exposure to maternal aABs affects the risk of a broader range of NDD during this neurodevelopmentally susceptible period. These findings contribute to our understanding of the etiology of AU and ID important for identification and intervention. Future studies on brain development as well as the mechanisms of action of gestational exposure to maternal aABs will allow us to determine how maternal aABs target cells in the developing brain and lead to altered neurodevelopment associated with these disorders.

## Figures and Tables

**Table 1:** Three-way patterns of maternal aABs that predict AU and ID risk identified by LASSO modeling.

Previously identified two aAB MARA patterns (7)	Novel Three-way Maternal aAB pattern	All N = 1144	AU N = 540 *N (%)*	ID N = 184 *N (%)*	GP N = 420 *N (%)*	OR AU vs. GP (N = 540)	OR ID vs. GP (N = 184)	OR GP (ref) (N = 420)
CRMP1 + STIP1	CRMP1 + STIP1+LDHA	6	6 (1.1%)	0 (0%)	0 (0%)	**4.44**	1.00	1.00
CRMP1 + GDA	CRMP1 + GDA+LDHA	7	7 (1.3%)	0 (0%)	0 (0%)	**4.15**	1.00	1.00
CRMP1 + CRMP2	CRMP1 + CRMP2+YBOX	1	1 (0.2%)	0 (0%)	0 (0%)	**1.95**	1.00	1.00
	CRMP1 + CRMP2+LDHA	8	8 (1.5%)	0 (0%)	0 (0%)	**5.30**	1.00	1.00
	CRMP1 + CRMP2+LDHB	4	4 (0.7%)	0 (0%)	0 (0%)	**1.56**	1.00	1.00
CRMP2 + STIP1	CRMP2 + STIP1+LDHA	2	2 (0.4%)	0 (0%)	0 (0%)	**1.05**	1.00	1.00
	CRMP2 + STIP1+LDHB	4	4 (0.7%)	0 (0%)	0 (0%)	**3.07**	1.00	1.00
	CRMP2 + STIP1+GDA	3	2 (0.4%)	1 (0.5%)	0 (0%)	**1.26**	**1.29**	1.00
STIP1 + NSE	STIP1 + NSE + GDA	3	3 (0.6%)	0 (0%)	0 (0%)	**3.39**	1.00	1.00
YBOX+LDHA, YBOX+LDHB, or YBOX + GDA	YBOX+LDHA+LDHB	2	2 (0.4%)	0 (0%)	0 (0%)	**3.24**	1.00	1.00
	YBOX+LDHA + GDA	2	2 (0.4%)	0 (0%)	0 (0%)	**3.92**	1.00	1.00
	YBOX+LDHB + GDA	1	1 (0.2%)	0 (0%)	0 (0%)	**1.95**	1.00	1.00
	YBOX+LDHA+STIP1	2	1 (0.2%)	1 (0.5%)	0 (0%)	1.00	**2.47**	1.00
	YBOX+LDHA + NSE	1	0 (0%)	1 (0.5%)	0 (0%)	1.00	**8.80**	1.00
	YBOX + GDA+NSE	1	1 (0.2%)	0 (0%)	0 (0%)	**1.95**	1.00	1.00
**De novo three-way patterns**	CRMP1 + NSE+LDHB	5	1 (0.2%)	4 (2.2%)	0 (0%)	1.00	**9.52**	1.00
	CRMP2 + NSE+LDHA	5	4 (0.7%)	0 (0%)	1 (0.2%)	**1.09**	1.00	1.00
	CRMP2 + GDA+LDHB	3	3 (0.6%)	0 (0%)	0 (0%)	**1.84**	1.00	1.00
	NSE+LDHA+LDHB	6	4 (0.7%)	1 (0.5%)	1 (0.2%)	**1.26**	1.00	1.00
	NSE+LDHA + GDA	10	8 (1.5%)	0 (0%)	2 (0.5%)	**1.55**	1.00	1.00
	NSE+LDHB + GDA	3	3 (0.6%)	0 (0%)	0 (0%)	**1.21**	1.00	1.00
	LDHA+LDHB + GDA	19	9 (1.7%)	1 (0.5%)	9 (2.1%)	**0.64**	**0.64**	1.00

**Table 2:** Three-way patterns of maternal aABs that predict AU ID and AU noID subgroup risk identified by LASSO modeling.

Previously identified two aAB MARA patterns (7)	Novel Three-way Maternal aAB pattern	All N = 924	AU ID N = 285	AU noID N = 219	GP N = 420	OR AU ID vs. GP (N = 540)	OR AU noID vs. GP (N = 184)	OR GP (ref) (N = 420)
CRMP1 + CRMP2	CRMP1 + CRMP2+YBOX	1	1 (0.4%)	0 (0%)	0 (0%)	**1.69**	1.00	1.00
	CRMP1 + CRMP2+LDHA	8	5 (1.8%)	3 (1.4%)	0 (0%)	**2.64**	**2.64**	1.00
CRMP1 + STIP1	CRMP1 + STIP1+NSE	1	1 (0.4%)	0 (0%)	0 (0%)	**1.15**	1.00	1.00
	CRMP1 + STIP1+LDHA	6	4 (1.4%)	2 (0.9%)	0 (0%)	**1.93**	**1.93**	1.00
CRMP1 + GDA	CRMP1 + GDA+LDHA	7	6 (2.1%)	1 (0.5%)	0 (0%)	**2.94**	1.00	1.00
CRMP2 + STIP1	CRMP2 + STIP1+LDHB	4	3 (1.1%)	1 (0.5%)	0 (0%)	**1.42**	**1.42**	1.00
STIP1 + NSE	STIP1 + NSE+LDHB	2	0 (0%)	2 (0.9%)	0 (0%)	1.00	**4.75**	1.00
YBOX+LDHA, YBOX+LDHB, or YBOX + GDA	YBOX + GDA+NSE	1	0 (0%)	1 (0.5%)	0 (0%)	1.00	**2.40**	1.00
	YBOX+LDHA+LDHB	2	1 (0.4%)	1 (0.5%)	0 (0%)	**1.25**	**1.25**	1.00
	YBOX+LDHA + GDA	1	0 (0%)	1 (0.5%)	0 (0%)	1.00	**2.40**	1.00
	YBOX+LDHB + GDA	1	0 (0%)	1 (0.5%)	0 (0%)	1.00	**2.40**	1.00
**De novo three-way patterns**	CRMP1 + NSE+LDHB	1	1 (0.4%)	0 (0%)	0 (0%)	**1.69**	1.00	1.00
	CRMP2 + GDA+LDHB	3	3 (1.1%)	0 (0%)	0 (0%)	**2.86**	1.00	1.00
